# Zebrafish Cyclin-Dependent Protein Kinase–Like 1 (*zcdkl1*): Identification and Functional Characterization

**DOI:** 10.3390/ijms12063606

**Published:** 2011-06-03

**Authors:** Li-Sung Hsu, Cyong-Jhih Liang, Chen-Yuan Tseng, Chi-Wei Yeh, Jen-Ning Tsai

**Affiliations:** 1 Institute of Biochemistry and Biotechnology, Chung Shan Medical University, Taichung 402, Taiwan; E-Mails: choch7211@msn.com (C.-J.L.); rockforever1102@hotmail.com (C.-Y.T.); n131716@hotmail.com (C.-W.Y.); 2 Clinical Laboratory, Chung Shan Medical University Hospital, Taichung 402, Taiwan; 3 School of Medical Laboratory and Biotechnology, Chung Shan Medical University, Taichung 402, Taiwan; E-Mail: zgsk3@hotmail.com

**Keywords:** cyclin dependent protein kinase-like 1 (*cdkl1*), expression pattern, floor plate, zebrafish

## Abstract

The cyclin-dependent protein kinase family regulates a wide range of cellular functions such as cell cycle progression, differentiation, and apoptosis. In this study, we identified a zebrafish cyclin-dependent protein kinase-like 1 protein called zebrafish *cdkl1* (*zcdkl1*), which shared a high degree of homology and conserved synteny with mammalian orthologs. *zcdkl1* exhibited abilities for phosphorylation of myelin basic protein and histone H1. RT-PCR analysis revealed that *zcdkl1* was expressed starting from fertilization and continuing thereafter. In adult tissues, *zcdkl1* was predominantly detected in brain, ovary, and testis, and was expressed at low levels in other tissues. At 50% epiboly stage, *zcdkl1* was widely expressed. At 12 to 48 h post-fertilization, *zcdkl1* was predominantly expressed in the hypochord, the medial and lateral floor plate, and the pronephric duct. Interference of *zcdkl1* expression resulted in abnormalities, such as brain and eye malformation, pericardial edema, and body axis curvature. Disruption of *zcdkl1* reduced *neurogenin-1* in the brain and *sonic hedgehog* expression in the floor plate region. These deformities were apparently rescued by co-injection of *zcdkl1* mRNA. Findings of this study indicate that *zcdkl1* plays an essential role in zebrafish development.

## 1. Introduction

The cyclin-dependent protein kinase (CDK) protein family, a group of serine/threonine kinases, has been demonstrated to be an important regulator of cell division at the G1/S and G2/M checkpoints [1]. Progression of the cell cycle depends on the association of CDK with a specific cyclin to form active complexes, which then control the expression of downstream genes involved in the cell cycle [1]. Interaction of cdc2 (CDK1) with cyclin A mediates the transition of the G2/M phase, whereas activation of cdc2 after interaction with cyclin B is the key step that triggers mitosis [2,3].

A family of protein kinases called cdc2-related kinase, which include CDK10, PCTAIRE, CDKL1, CDKL2, and CDKL3, was recently identified through the biochemical and genetic approach; they are named based on the sequence corresponding to the PSTAIRE motif of cdc2 [4]. Each cdc2-related kinase contains a distinct expression pattern and performs unique biological functions. Mammalian CDKL1 is predominantly expressed in the brain, lungs, kidneys, and ovaries [4,5], and its up-regulation has been found in glial cells during gliosis [6]. CDKL1 is highly expressed in rat neuroblastoma cells but weakly expressed in normal neuron cells [6]. Using specific antibodies against CDKL1, Kim, *et al.* demonstrated that CDKL1 protein levels increased during postnatal development of the heart in rats [7].

Zebrafish has now become a widely used animal model in the study of genetics and developmental biology [8]. However, the importance of cdc2-related kinases in the development of zebrafish still needs to be elucidated. In the present study, we identified and characterized the role of *zcdkl1* in zebrafish development.

## 2. Results

### 2.1. Identification, Genomic Structure, and Synteny Analysis of *zcdkl1*

**Figure 1:**
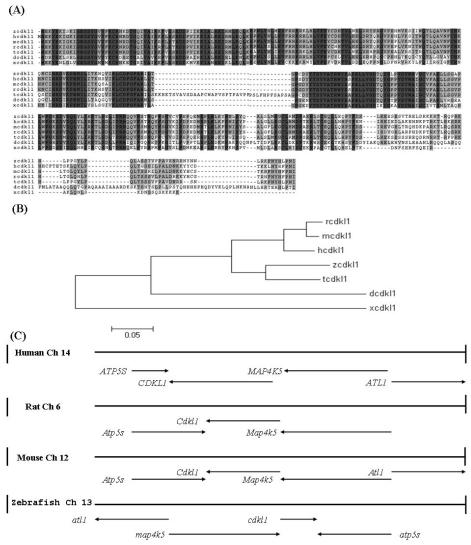
(**A**) Multiple alignment of *zcdkl1* amino acid sequences with those of other vertebrates. *CDKl1* from different species were aligned using the ClustalW program. Identical sequences are shaded in black, while residue similarities over four CDKL1 proteins are denoted in gray; (**B**) A phylogenetic tree was constructed based on multiple alignments of CDKL1 proteins using the MegAlign program in DNASTAR package with the neighbor joining method. Scale bars indicate nucleotide substitutions (×100); (**C**) A graphical representation of conserved synteny of the *cdkl1* gene clusters in human chromosome 14, rat chromosome 6, mouse chromosome 12, and zebrafish chromosome 13. Ch denotes the chromosome. A dashed line denotes that this gene was predicted by computer annotation.

A prior DNA microarray analysis revealed the downstream targets of zebrafish *regulator of G protein signaling 7* (*zrgs7*). One putative protein kinase (zgc 101002, accession number XM_696464) was found to be slightly down-regulated in *zrgs7* knockdown morphants and was selected for further investigation. This gene shared high homology with human cyclin-dependent protein kinase-like 1 (CDKL1) and was termed zebrafish *cdkl1* (*zcdkl1*). The *zcdkl1* gene contained 1525 nucleotides encoding an open reading frame of 350 amino acids. The deduced protein sequence of *zcdkl1* contained 11 characterized protein kinase subdomains that were localized in the *N*-terminal region. The conserved sequence KKIALRE was found at 45 to 51 aa. Homology of the deduced amino acids sequences with that of other species was analyzed using the CLUSTAL W program (Figure 1A). The *zcdkl1* protein shared 85%, 77%, 76%, 54%, and 50% identity with the cdkl1 of *Tetraodon*, human, mouse, *Drosophila*, and *Xenopus*, respectively. The kinase domain displayed more than 90% identity, whereas the variable region was localized in the *C*-terminal domain. The three regulatory phosphorylation sites Ser^14^, Tyr^15^, and Thr^159^ corresponding to Thr^14^, Tyr^15^, and Thr^161^ of human CDK1 were also found in *zcdkl1*. Moreover, *zcdkl1* contained conserved MAP kinase activation motif, Thr^158^-Asp^159^-Tyr^160^, localized in the activation loop of subdomain VIII. Phylogenetic analysis based on the deduced amino acids revealed that *zcdkl1* was clustered into a subclade with *Tetraodon cdkl1* (Figure 1B). This teleost fish branch is grouped closely with the mammalian branch consisting of humans, rats, and mice, whereas xcdkl1 formed a branch by itself.

To determine whether the *zcdkl1* gene shared conserved synteny with mammalian species, we compared zebrafish linking group (LG) 13 with human chromosome 14, mouse chromosome 12, and rat chromosome 6, where CDKL1 is localized. The *zcdkl1* gene clustered with *atlastin GTPase 1*, *mitogen-activated protein kinase kinase kinase kinase 5*, and *ATP synthase* in LG13. This suggests conserved synteny with genes in human chromosome 14, rat chromosome 6, and mouse chromosome 12, although with an inverted gene order (Figure 1D). Our findings indicate that *zcdkl1* is a true ortholog of mammalian *CDKL1*.

### 2.2. *zcdkl1* Phosphorylates Myelin Basic Protein and Histone H1

**Figure 2:**
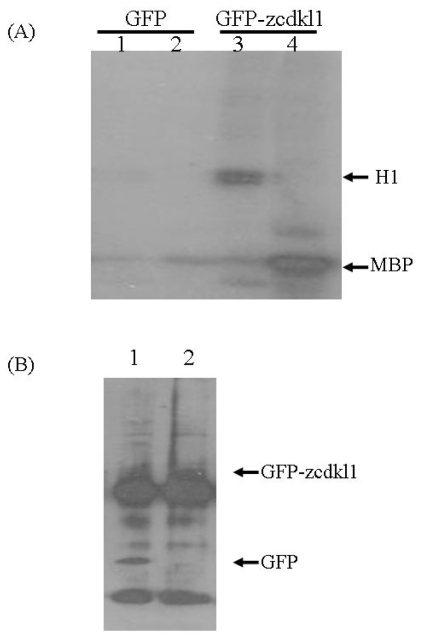
Kinase activity assay of zcdkl1 in HEK 293 cells transfected with pEGFP or pEGFP-*zcdkl1*. Twenty-four h post-transfection, cells were lysed with lysis buffer and subjected to immunoprecipitation with anti-GFP antibody. (**A**) Immunoprecipitated complexes underwent *in vitro* kinase assay using myelin basic protein (MBP) and histone H1 as substrates. Phosphorylation of histone H1 (lane 1 and 3) and MPB (lanes 2 and 4) was detected by autoradiography; (**B**) Immunoprecipitated complexes (lane 1 for GFP alone; lane 2 for GFP-*zcdkl1*) were subjected to Western blot analysis using anti-GFP antibodies.

Previous studies have demonstrated that human CDKL1 is capable of autophosphorylation and can phosphorylate myelin basic protein (MBP) and histone H1 [5,6]. To investigate the activity of *zcdkl1*, we transfected HEK 293 cells with green fluorescent protein (GFP) alone or GFP-tagged *zcdkl1* and performed an immunoprecipitation protocol using anti-GFP antibodies. Immunoprecipitated complexes were subjected to *in vitro* kinase assay. Figure 2 shows that phosphorylation of histone H1 and MBP was found in immunoprecipitates from cells transfected with pGFP-*zcdkl1* but not in those transfected with pEGFP alone, although very weak autophosphorylation was detected.

### 2.3. Temporal and Spatial Expression Pattern of *zcdkl1* RNA Transcript in Developmental and Adult Tissues

**Figure 3:**
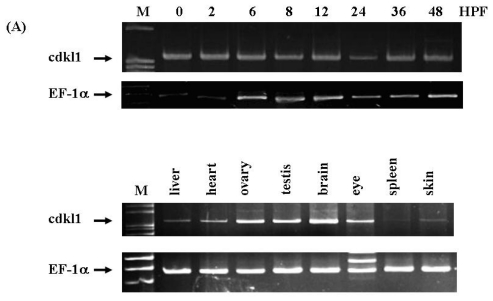

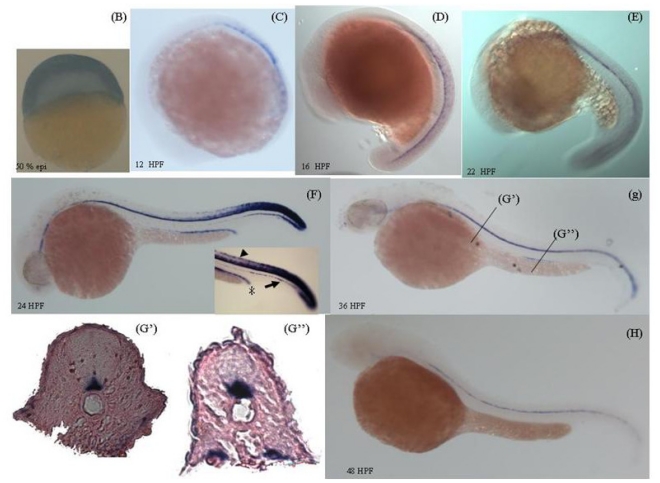
Temporal and spatial expression of *zcdkl1* gene. (**A**) Total RNA derived from different stages of development (upper panel) and adult tissues (lower panel) were converted into cDNA and subjected to PCR amplification. PCR fragments were separated by electrophoresis on 3% agarose gel and visualized by ethidium bromide staining. Elongation factor 1 α was included as internal control. (**B**–**H**) Expression of *zcdkl1* was analyzed in whole mount *in situ* hybridization. The developmental stage is indicated in the lower left (G′ and G″), while the transverse section is indicated by line in panel (G). Sections were counterstained with eosin Y. (B–H) Lateral view with the anterior pole to the left. Insert in (F) represents the caudal region with higher magnification. The arrowhead indicates the dorsal neurons in the spinal cord. The arrow indicates the hypochord. The asterisk indicates the pronephric duct.

To gain insight into the spatial expression pattern of *zcdkl1*, cDNA from different stages of developmental and adult tissues were used for semi-quantitative RT-PCR. Results indicated that *zcdkl1* RNA transcripts were expressed immediately after fertilization and was continuously expressed thereafter. In adult tissues, *zcdkl1* was found to be abundantly expressed in the brain, ovary, and testes. Lower expression levels were found in the liver and heart, whereas very weak expression was detected in the skin and spleen (Figure 3A).

In order to elucidate the expression pattern of *zcdkl1*during development, we performed whole-mount *in situ* hybridization using the antisense riboprobe of *zcdkl1*. As shown in Figure 3B, *zcdkl1* transcripts were diffusedly expressed at 50% epiboly. *zcdkl1* mRNA transcripts were predominantly expressed in the floor plate region and were weakly expressed in the dorsal neurons at 12 and 16 h post-fertilization (HPF). At 24 HPF, we found high levels of *zcdkl1* expression in the floor plate, dorsal neuron tube, tail bud, and pronephric duct, while low expression was found in the hypochord. At 36 HPF, *zcdkl1* expression was observed in the pronephric duct and floor plate and was weakly observed in the hypochord. RNA transcripts of z*cdk1* were expressed predominantly in the floor plate at 48 HPF. Samples of the transected section at 36 HPF indicated that *zcdkl1* transcripts were detected in both medial and lateral floor plate cells (Figure 3B).

### 2.4. Knockdown of *zcdkl1* Induced Malformation of Zebrafish

**Figure 4:**
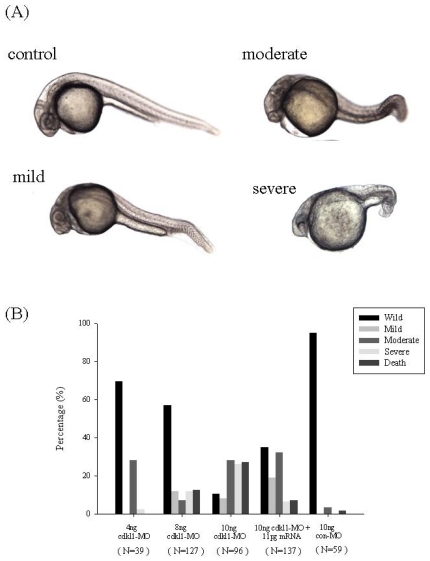
Knockdown of *zcdkl1* produced defective phenotypes. Embryos at 1 to 4 cells stages received different dosages of morpholino. (**A**) Phenotype of *zcdkl1* morphant at 24 HPF. Lateral view with the anterior pole to the left; (**B**) Percentages of defective phenotype produced by different dosages of *zcdkl1* MO, 10 ng *zcdkl1* MO plus 11 pg *zcdkl1* mRNA, and 10 ng control MO. N value denotes the total injected embryos from three independent experiments.

To investigate the development role of *zcdkl1*, we performed microinjection of specific morpholino antisense oligonucleotides complementary to the translation start site of *zcdkl1*. Morphant phenotypes were divided into three classes according to axis malformation. Small head and eyes, slightly shorter trunk, and bent tail were found in the mild phenotype, whereas the moderate phenotype presented with opaque regions in the brain, pericardial edema, shorter and curved trunk, and kinked tail. Malformation of the brain and eye region and very short trunk were observed in the severe type of morphants (Figure 4A). The percentage of deformation and death among morphants was proportional to the amount of *zcdkl1* morpholino injected (Figure 4B).

We performed an RNA rescue experiment to confirm that the characterized phenotypes were indeed caused by the down-regulation of *zcdkl1* expression. No distinguished phenotype was found in embryos that received *zcdkl1* mRNA. The abnormal phenotypes of the morphants were noticeably rescued by co-injection of *zcdkl1* mRNA with morpholino (Figure 4B). Application of 11 pg of *zcdkl1* mRNA not only significantly reduced death and severity of morphants from 26.5% to 7.3% and 16.8% to 6.5%, but also increased mild and wild type rates from 13.8% to 19% and 16.9% to 35.1%., respectively. Our results indicate that knockdown of *zcdkl1* caused abnormalities in zebrafish development that can be apparently recovered by co-injection of *zcdkl1* mRNA.

**Figure 5:**
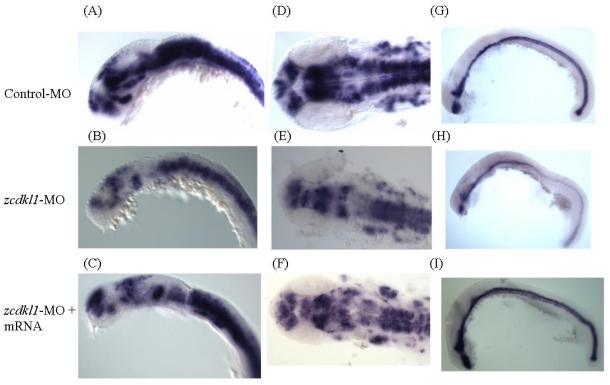
Expression pattern of neurogenin 1 (*ngn1*) and sonic hedgehog (*shh*) in *zcdkl1* morphants. Morphants that received 10 ng control MO (**A**, **D**, and **G**), 10 ng cdkl1 MO (**B**, **E**, and **H**), and 10ng cdkl1 MO + 11 pg cdkl1 mRNA (**C**, **F**, and **I**) were subjected to whole mount *in situ* hybridization using *ngn1* (A–F) and *shh* (G–I) riboprobes. Lateral (A–C and G–I) and dorsal (D–F) views with the anterior pole to the left.

To further characterize the effect of zcdkl1 knockdown on neuron development, we performed *in situ* hybridization using riboprobe for *neurogenin-1* (*ngn1*), a neuron cell marker. We chose stage-matched embryos for comparison of expression pattern to avoid delayed development. Figure 5 shows reduced *ngn1* signals at the forebrain, anterior midbrain, and cranial ganglia in embryos that received 10 ng MO at 24 hpf. Co-injection with *zcdkl1* MO and *zcdkl1* mRNA led to recovery of the *ngn1-*positive cells.

Moreover, we determined the effects of *zcdkl1* knockdown on floor plate formation through *in situ* hybridization using sonic hedgehog (*shh*) antisense riboprobes in morphants. *shh* signal was attenuated in notochord, floor plate region, and tail bud of *zcdkl1* morphants. Interestingly, stronger *shh* expression pattern was found in embryos co-injected with *zcdkl1* MO and mRNA (Figure 5).

## 3. Experimental Section

### 3.1. Maintenance of Zebrafish

Zebrafish (*Danio rerio*) AB strain were raised and maintained at 28 °C on a 14 h light/10 h dark cycle according to a previously described method [9]. Developmental embryos were also staged according to a previously described method [10].

### 3.2. RT-PCR of *zcdkl*

Total RNA was purified from different stages and various tissues using TRIzol Reagent (Invitrogen) as recommended by the manufacturer. RT-PCR amplification was performed using 1 μL cDNA as the template with designed primers (sense: 5′-CGTATAGGAGATGCTGGCAG-3′ and anti-sense: 5′-ATGCAGTGAACGGTAGCTCTC-3′). Negative control using water instead of cDNA was concomitantly performed. The amplification of the elongation factor 1α was used as an internal control.

### 3.3. Plasmid Construction

The full coding region of z*cdk1* was amplified and subjected to ligate into a pGEMT-easy vector (Promega) and transformed to JM109 competent cells. pEGFP-*zcdk1* and pCS2-*zcdk1* were constructed by enzyme digestion and ligation into pEGFP-C1 and pCS2, respectively. All the plasmids were sequenced from both directions by an ABI 3100 DNA sequence (MISSION BIOTECH).

### 3.4. Whole Mount *in situ* Hybridization

The anti-sense riboprobes for *zcdkl1* were made by using in vitro transcription by T7 RNA polymerase in the presence of digoxigenin-labeled UTP and Nco I-digested linear DNA as template, following the manufacturer’s standard protocol (Roche). Whole-mount *in situ* hybridization was performed as previously described [9].

### 3.5. Morpholino Injection and mRNA Rescue

Morpholino (MO) antisense oligonucleotide (Gene-Tools) targeted against *zcdkl1* was designed as the following sequence: 5′-TGATCTTCTCATACTTCTCCATCGC-3′ corresponded to −3 to +22 of *zcdkl*. Control MO: 5′-CCTCTTACCTCAGTTACAATTTATA-3′ corresponding to the human β-globin gene was conducted as negative control. The MO was dissolved in 1 X Danieau solution containing 0.5% phenol Red and injected into embryos at 1 to 4 cells stage. For the mRNA rescue, capped RNA encoding full-length of transcripts of *zcdkl1* was synthesized from Not I-cutted pCS2-*zcdkl1* by using mMESSAGE mMACHINE kit (Ambion) according to the manufacturer’s recommendation. Morpholino was co-injected with 11 pg mRNA into 1 to 4 cell embryos.

### 3.6. Cell Culture and Transfection

Human embryo kidney HEK293 cells were maintained in an MEM medium supplemented with 10% fetal horse serum, 100 μg/mL streptomycin, and 100 U/mL penicillin. The cells were cultured in a humidified atmosphere of 95% air and 5% CO_2_. Plasmid DNA was transfected into HEK 293 using Lipofetamine reagent (Invitrogen). Twenty-four h after transfection, the cell lystaes were collected.

### 3.7. Immunoprecipitation and Kinase Activity of *zcdkl1*

Human HEK293 cells were transfected with pEGFP or pEGFP-*zcdk1*. Forty-eight hours post-transfection, the cells were lysed with a lysis buffer as previously described [11]. Three hundred μg cell lysates were subjected to perform immunoprecipitation using an anti-green fluorescent protein (GFP) antibody (Santa Cruz Biotechnology). After being washed three times with a kinase buffer (25 mM Tris-HCl, pH 7.5, 2 mM dithiothreitol, 0.1 mM sodium orthovanadate, and 10 mM MgCl_2_), the precipitates were suspended in 10 mL of kinase buffer containing 0.1 mM ATP, 5 μg histone H1 or myelin basic protein, and 5 μCi of [γ-^32^P] ATP. The reaction was incubated at 37 °C for 10 min and terminated by boiling in an SDS-PAGE sample buffer followed by electrophoresis on a 10% SDS-polyacrylamide gel. Phosphorylation was determined by autoradiography of the dried gel.

### 3.8. Western Blot Analysis

The immunoprecipitated complexes were obtained and separated by a 10% polyacrylamide gel, and then transferred to a nitrocellulose membrane. The membrane was blocked with phosphate buffer saline (PBS) containing 5% non-fat milk. Afterward, the membrane was incubated with mouse anti-GFP antibodies (Santa Cruz Biotechnology, USA) at 4 °C overnight. The membrane was washed with PBS containing 0.1% Tween-20, followed by reaction with an HRP-conjugated goat anti-mouse IgG antibody (Santa Cruz Biotechnology, USA). The signals were detected by an enhanced chemiluminescence kit (Amersham Pharmacia Biotech, UK).

## 4. Discussion

In this study, we investigated the expression pattern and biological role of *zcdkl1* in the development of zebrafish. The *zcdkl1* gene shared high homology with mammalian orthologs and had conserved synteny with inverted DNA order. *zcdkl1* exhibited phosphotransfer activity for the phosphorylation of MBP and H1 histone. RNA transcripts of *zcdkl1* were predominantly expressed in the floor plate, pronephric duct, and hypochord. Small brain and eyes and curved axes were found in *zcdkl1* morphants, but co-injection with *zcdkl1* mRNA partially rescued these phenotypes. Down-regulation of *zcdkl1* led to decreased *ngn-1* and *shh* expression in the floor plate, which can be rescued by co-injection with *zcdkl1* mRNA. To our knowledge, this is the first study to demonstrate the involvement of a cdk-related kinase in floor plate formation.

Expression of *zcdkl1* was found in both medial and lateral floor plates, extending from the zona limitans to the tail bud region. The floor plate consists of a group of non-neuronal cuboidal epithelial cells localized in the ventral midline to the neuron tube [12]. The zebrafish floor plate can be subdivided into the medial and lateral floor plates, which exhibit different development mechanisms and express specific gene markers [13]. *shh* signaling is necessary for specification of the lateral floor plate, while cyclops signaling plays a pivotal role in medial floor plate development [13–15]. Disruption of SHH signal leads to malformation of the floor plate, whereas s*hh* overexpression induces additional floor plate formation [16,17]. Moreover, s*hh* RNA transcripts are expressed in the notochord, floor plate, and anterior fin mesoderm [18]. Overlapping expression patterns of *shh* and *zcdkl1* suggests functional dependence of these two genes. Most strikingly, interference of *zcdkl1* expression slightly attenuated *shh* expression in the floor plate, whereas co-injection with *zcdkl1* mRNA rescued this phenomenon. Recently, T box-related homeodomain protein (tbx) and Foxa2 transcript factor were shown to bind to the floor plate enhancer region of *shh* and subsequently cooperated to regulate s*hh* expression in the floor plate [19]. foxa2 has been demonstrated to be capable of phosphorylating serine residues by casein kinase I (CKI). Nonetheless, CKI did not influence foxa2 transcription activity [20]. Scansite analysis revealed that two serine types (Ser 215 and Ser 302) localized in the DNA-binding domain and near the transcriptional activation domain can be phosphorylated by a proline-directed kinase such as cdc2 or cdk5. Since there are a number of cdc2-related kinases, CDKL1 has also been shown to be a proline-directed protein kinase that phosphorylates peptides containing the X-Ser-Pro-X motif [6]. Whether *zcdkl1* could phosphorylate foxa2 and subsequently increase foxa2 transcriptional activity, leading to *shh* expression, is still under investigation. This theory warrants further investigation.

Attenuated *ngn-1* expression in the forebrain, midbrain, and cranial ganglia was found in *zcdkl1* morphants. A previous report demonstrated that SHH triggers neurogenin-1 expression in primary cultured mouse trigeminal neural crest cells, which then promoted cell differentiation into sensory neurons. This effect was diminished in the presence of cyclopamine, an SHH-specific inhibitor [21]. Our findings revealed that disruption of *zcdkl1* expression may lead to *shh* attenuation and its downstream target expression.

## 5. Conclusion

In this study, we identified a protein kinase termed *zcdkl1* that shares high homology with mammalian orthologues. *zcdkl1* mRNA transcripts were predominantly expressed in the floor plate, hypochord, and pronephric ducts by whole mount *in situ* hybridization. Knockdown of z*cdkl1* in zebrafish resulted in deformities of the brain and eyes and abnormal axis formation. Reduction of *ngn-1* and *shh* expression were observed in *zcdkl1* morphants, which can be rescued by co-injection with *zcdkl1* mRNA. Findings suggest that *zcdkl1* plays a pivotal role in zebrafish development.
